# The Role of Proprotein Convertases in the Regulation of the Function of Immune Cells in the Oncoimmune Response

**DOI:** 10.3389/fimmu.2021.667850

**Published:** 2021-04-29

**Authors:** Mélanie Rose, Marie Duhamel, Franck Rodet, Michel Salzet

**Affiliations:** Université Lille, Inserm, CHU Lille, U1192, Laboratoire Protéomique, Réponse Inflammatoire et Spectrométrie de Masse (PRISM), Lille, France

**Keywords:** proprotein convertase, microbiota, oncoimmune, macrophages, immunothearpy, furin, proprotein convertase 1/3

## Abstract

Proprotein convertases (PC) are a family of 9 serine proteases involved in the processing of cellular pro-proteins. They trigger the activation, inactivation or functional changes of many hormones, neuropeptides, growth factors and receptors. Therefore, these enzymes are essential for cellular homeostasis in health and disease. Nine PC subtilisin/kexin genes (PCSK1 to PCSK9) encoding for PC1/3, PC2, furin, PC4, PC5/6, PACE4, PC7, SKI-1/S1P and PCSK9 are known. The expression of PC1/3, PC2, PC5/6, Furin and PC7 in lymphoid organs such as lymph nodes, thymus and spleen has suggested a role for these enzymes in immunity. In fact, knock-out of *Furin* in T cells was associated with high secretion of pro-inflammatory cytokines and autoantibody production in mice. This suggested a key role for this enzyme in immune tolerance. Moreover, Furin through its proteolytic activity, regulates the suppressive functions of Treg and thus prevents chronic inflammation and autoimmune diseases. In macrophages, Furin is also involved in the regulation of their inflammatory phenotype. Similarly, PC1/3 inhibition combined with TLR4 stimulation triggers the activation of the NF-κB signaling pathway with an increased secretion of pro-inflammatory cytokines. Factors secreted by PC1/3 KD macrophages stimulated with LPS exert a chemoattractive effect on naive auxiliary T lymphocytes (Th0) and anti-tumoral activities. The link between TLR and PCs is thus very important in inflammatory response regulation. Furin regulates TL7 and TLR8 processing and trafficking whereas PC1/3 controls TLR4 and TLR9 trafficking. Since PC1/3 and Furin are key regulators of both the innate and adaptive immune responses their inhibition may play a major role in oncoimmune therapy. The role of PCs in the oncoimmune response and therapeutic strategies based on PCs inhibition are proposed in the present review.

## Introduction

The proteome is illustrated by its great complexity. Each protein can undergo post-translational modifications which regulate their function, location, activity and structure. In this way, proteolytic cleavage regulates many biological processes through the modulation of functional maturation and bioavailability of many cellular and secreted proteins. Proteolysis is carried out by proteases classified in 5 families according to their sequence homologies: serine proteases, metalloproteases, cysteine proteases, threonine proteases and aspartic acid. Serine proteases are the largest proteases family in human (1). Among them, proprotein convertases (PCs) are involved in the processing of cellular pro-proteins. They trigger the activation, inactivation or functional changes of many hormones, neuropeptides, growth factors and receptors. Therefore, these enzymes are essential for cellular homeostasis in health and disease. Nine PC subtilisin/kexin genes (PCSK1 to PCSK9) encoding for PC1/3, PC2, furin, PC4, PC5/6, PACE4, PC7, SKI-1/S1P and PCSK9 are known (2). The first seven enzymes are very close and cleave protein precursors according to the pattern (R/K)X_n_(R/K)↓. SKI-1 activates membrane transcription factors by proteolysis according to the RX(L/V/I)X↓ pattern. Finally, PCSK9 achieves a catalytic autocleavage after its VFAQ↓ residue (2). PCs are synthesized as inactive precursors. They must undergo proteolytic cleavage to reach their active form (3). PCs show tissue-specific expression except for PC7, SKI-1 and furin which have ubiquitous expression (2). PCSK9 is thus found in the liver, intestines and kidneys (4). PC5/6 is expressed in the intestine, kidneys and ovaries and PACE 4 in the muscles, heart, pituitary, cerebellum and kidneys (2). PC4, PC1/3 and PC2 have a more specific tissue distribution. PC4 is expressed in the germ cells and the placenta (5). As for PC1/3 and PC2, they are mainly found in the neuroendocrine system (2) and have been more recently described in the immune system (6). **Table 1** summarizes the tissue distribution and substrates of PCs as well as the description of knock out PCs models (14, 15). The expression of PC1/3, PC2, PC5/6, furin and PC7 under LPS stimulation in immune cells and lymphoid organs suggested a role for these enzymes in immunity (6, 16). This review focuses on the roles of PCs in immune cells (**Table 2**) and their possible use in oncoimmune therapeutic strategies.

**Table 1:** Tissue distribution of PCs and their substrates and phenotypic description of knock out PCs models.

Proprotein Convertase - *Gene*	Substrate example	Expression	KO models
**PC1/3 - *Pcsk1*** **PC2 - *Pcsk2***	POMC, proinsuline, proglucagon	Neuroendocrine tissus	Dwarfism (7) Growth disrupting (7, 8)
**Furin - *Furin***	TGF-β, BMP10	Ubiquitary	Embryonic lethality Heart and bone malformation (9, 10)
**PC4 - *Pcsk4***	IGF2, PACAP, ADAM	Placenta Gonads	Reduced fertility (11)
**PC5/6 - *Pcsk5***	GDF11	Intestine, kidneys and ovaries	Lethality at birth Defect in cytoskeletal development (12)
**PACE4 - *Pcsk6***	TGF-β, BMP, ADAM	Muscles, heart, pituitary, cerebellum and kidneys	Lethality Heart and bone malformation (13)
**PC7 - *Pcsk7***	Transferin receptor	Ubiquitary	No abnormality (14)
**SKI-1 - *Mbtps1***	SREBPs	Ubiquitary	Embryonic lethality (14)
**PCSK9 - *Pcsk9***	PCSK9	Liver, intestines and kidneys	Increase in LDLR expression, decrease in circulating cholesterol (14)

**Table 2:** Summary of functions of PCs in the immune system.

	Furin	PC1/3	PC7
**Mφ**	Anti-inflammatory orientation	
		Pro-tumoral orientation
**Treg**	Immunosuppressive activity		
**T cell**	Anti-inflammatory response		
**B cell**	Negative regulation of Ig secretion		
**Other global immune functions**	TLR7, 8, 9 processing	TLR4, 9 trafficking	TLR7 processing
	Antigenic peptides cleavage		Stable MHC-I expression

## Adaptive Immunity – Lymphocytes

In mice and human Th1 lymphocytes, treatment with IL-12 induced the over-expression of furin in a Stat4-dependent manner. In these cells, furin also regulates IFN-γ production (17). Embryonic lethality caused by furin deletion restricts *in vivo* studies. However, the cell-specific deletion of furin allows the study of its role in T cells without lethality. Thus, T cells specific deletion of furin did not induce any major developmental defects and resulted in a viable mouse model. These mice showed evidence of inflammation and fibrosis. In T cells, inhibition of furin did not disturb their development but considerably reduced their ability to secrete the anti-inflammatory cytokines transforming growth factor beta 1 (TGF-β1) and IL-10. The absence of furin was also associated with increased secretion of pro-inflammatory cytokines such as IL-6 and IFN-γ. Positive regulation of several genes generally associated with activation of T cells such as Fos, Jun and IFN-γ was also observed (18). Furin was also involved in B cell activation as demonstrated by higher levels of serum immunoglobulins after furin inhibition. Altogether, these results revealed a key role for this enzyme in immune tolerance (18).

Regulatory T cells (Treg) are central in the maintenance of immune tolerance (19). The transcription factor Foxp3 is fundamental in the regulation of Treg functions. Potential cleavage sites for PCs were identified in Foxp3 protein sequence and proteolytic activity of PC1/3 and PC7 on Foxp3 was observed in mice Treg. Moreover, IL-10 secretion was increased in Treg over-expressing the Foxp3 truncated form. These observations demonstrated that PC1/3 and PC7 controlled Treg immunosuppressive activity through Foxp3 cleavage (19). Unfortunately, these statements have not been confirmed in human yet (20). Indeed, the involvement of PCs in Foxp3 cleavage and its biological significance remain to be determined. The anti-inflammatory cytokine TGF-β1 is also involved in the maintenance of peripheral tolerance and protection against autoimmune diseases. TGF-β1 is synthesized as an inactive substrate (proTGF-β1) and must be cleaved to generate the mature and biologically active cytokine. Several studies have demonstrated that furin was essential for the maturation of proTGF-β1 in Treg (18, 21). Altogether, these observations suggest that PCs, through their proteolytic activities, regulate the suppressive functions of Treg. This may prevent chronic inflammation and autoimmune disease.

## Innate Immunity – Macrophages

Furin is also implicated in the regulation of macrophages activity. Indeed, furin-deficient macrophages showed positive regulation of many genes involved in their activation such as Serpinb1a, Serpinb2, Il6, Il1-β, Ccl2, and Ccl7 (22). An over-expression of pro-inflammatory cytokines such as TNF-α and Il-6 was also observed. On the contrary, the anti-inflammatory cytokine IL-10 was under-expressed. Besides, the inhibition of furin combined with LPS or IFN-γ stimulation resulted in an over-expression of Nos2, a pro-inflammatory phenotype marker. In contrast, the expression of Arg1, an anti-inflammatory phenotype marker was reduced (22). Thus, furin is also involved in the regulation of the inflammatory phenotype of macrophages by promoting an anti-inflammatory orientation.

**Figure 1:**
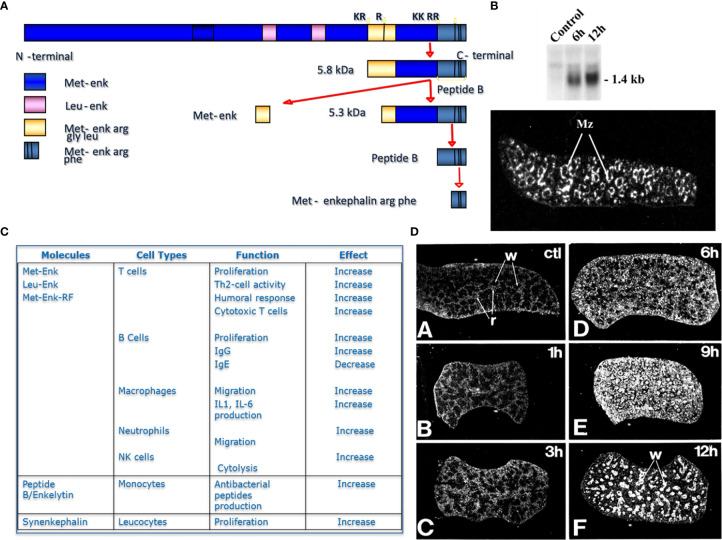
Proenkephalin and proprotein convertases in immune cells. **(A)** Scheme of Proenkephalin cleavage by PCs in macrophages, **(B)** Expression of proenkephalin in spleen with or without LPS treatment in time course, inset photograph representing the expression of proenkephalin in spleen ‘marginal zone after 12h LPS treatment, **(C)** Table of the immune action of the enkephalins on immune cells, **(D)** identification of PC1/3 expression in rat spleen in time course after LPS treatment.

**Figure 2:**
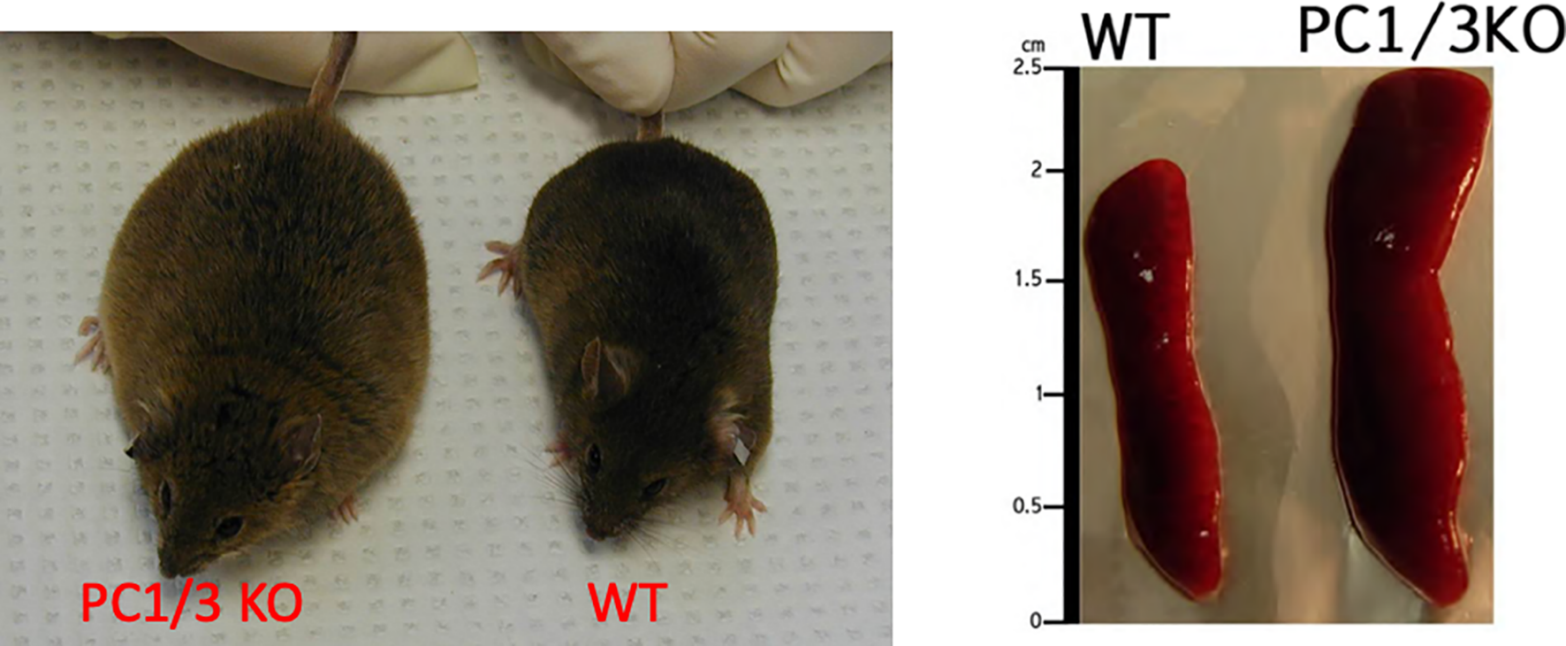
Comparison of PC1/3 KO mice phenotype with WT and the size of their spleen.

Similar results were observed after inhibition of PC1/3 in macrophages. PC1/3 has been originally described in the neuroendocrine system for its involvement in the processing of hormone and neuropeptide precursors. However, some studies have demonstrated that several neuropeptides precursors such as proenkephalin, pro-opiomelanocortin or chromogranins were expressed in macrophages (23, 24). Neuropeptides such as enkephalins or antimicrobial peptides (peptide B, enkelytin) were involved in immune response regulation (25). These peptides were processed by PC1/3 in immune cells, especially macrophages (6) (**Figure 1**). These observations led to more detailed studies to decipher the role of PC1/3 in immunity. For that purpose, the PC1/3 KO mouse model was used. These mice displayed enlarged spleens and were dwarf (**Figure 2**). Macrophages isolated from these mice had an increased secretion of pro-inflammatory cytokines (26). Following injection of LPS the resulting cytokine storm induced a septic shock (26). These observations were confirmed on rat alveolar macrophage NR8383 cell line in which the expression of PC1/3 has been knocked down (PC1/3 KD) (27). Proteomic analyses performed on these cells described more precisely how PC1/3 regulated their phenotype and activity. They revealed that PC1/3 inhibition led to a complete cytoskeleton reorganization and combined with TLR4 stimulation to a longer activation of the NF-κB signaling pathway (28). A similar result was observed following TLR9 activation (29). Thus, following TLR4 or TLR9 stimulation, PC1/3 KD macrophages also secreted more pro-inflammatory cytokines such as TNF-α, IL-1α and IL-1β (28). On the contrary, the activation of the anti-inflammatory STAT3 signaling pathway was reduced (28). Interestingly, factors secreted by PC1/3 KD macrophages stimulated with LPS exerted a chemoattractive effect on naive auxiliary T lymphocytes (Th0) (28). Of note, these secretomes also contained factors displaying anti-tumoral activities (28). Moreover, epigenetic studies established that PC1/3 KD macrophages exhibited a high level of methylation of H3K27 and of H3K79. Mono-, di-, and trimethylations were also detected, especially for H3K27. Under IL-10 treatment, H3K27 methylation fell, whereas H3K79me2, H3K9ac and H3K18ac remained high. Histone acetylation generally correlates with gene activation. PC1/3 KD macrophages treated with IL-10 also expressed factors involved in chemokine signaling pathway and pro-inflammatory factors such as Klhl29, Uba7, Ntpcr, and Dennd4c (30). Altogether, these results show that the inhibition of PC1/3 through shRNA (31) or peptidomimetic inhibitor (32) orientates macrophages towards a pro-inflammatory and anti-tumoral phenotype.

## TLRs Signaling and PCs

To recognize a large number of pathogen-associated molecular patterns and perform their immunological functions, immune cells express receptors such as Toll-like receptors (TLRs). TLRs are essential for the discrimination between self and non-self and promote innate and adaptive immune responses (33). Several studies have shown that PCs regulated the functions of TLRs to control the activity of immune cells. In this context, it was demonstrated that the antiviral activity of TLR7 in human THP1 macrophages, B cells and dendritic cells depended on its proteolytic cleavage by furin in the endosomes (34). Indeed, several potential cleavage sites for PCs were identified within the TLR7 protein sequence. Mutations in these sequences decreased the proportion of truncated TLR7 and therefore the cell response to TLR7 agonists. In human THP1 macrophages, *furin* and *pc7* knock down also resulted in marked inhibition of TLR7 processing. The same results were also depicted after inhibition of furin, PACE4, PC5/6 and PC7 by dicoumarol derivatives DC1 and DC2. Inhibition of TLR7 cleavage was also observed in LoVo cells expressing the preprosegments of PC5/6 and PC7. These preprosegments acted as specific inhibitors of the respective mature PCs. These studies revealed that TLR7 cleavage could be performed by several PCs family members. Using DC1, the requirement of furin-like PCs for TLR7 signaling was also demonstrated in human blood pDCs and B cells. In these cells, inhibition of furin-like PCs also decreased the cell responses to TLR7 and TLR9 agonists suggesting their involvement in TLR9 processing (34). In the same way, a potential proprotein convertase recognition site was observed in TLR8 sequence and furin inhibition in macrophages reduced their response to TLR8 agonist (35).

**Figure 3:**
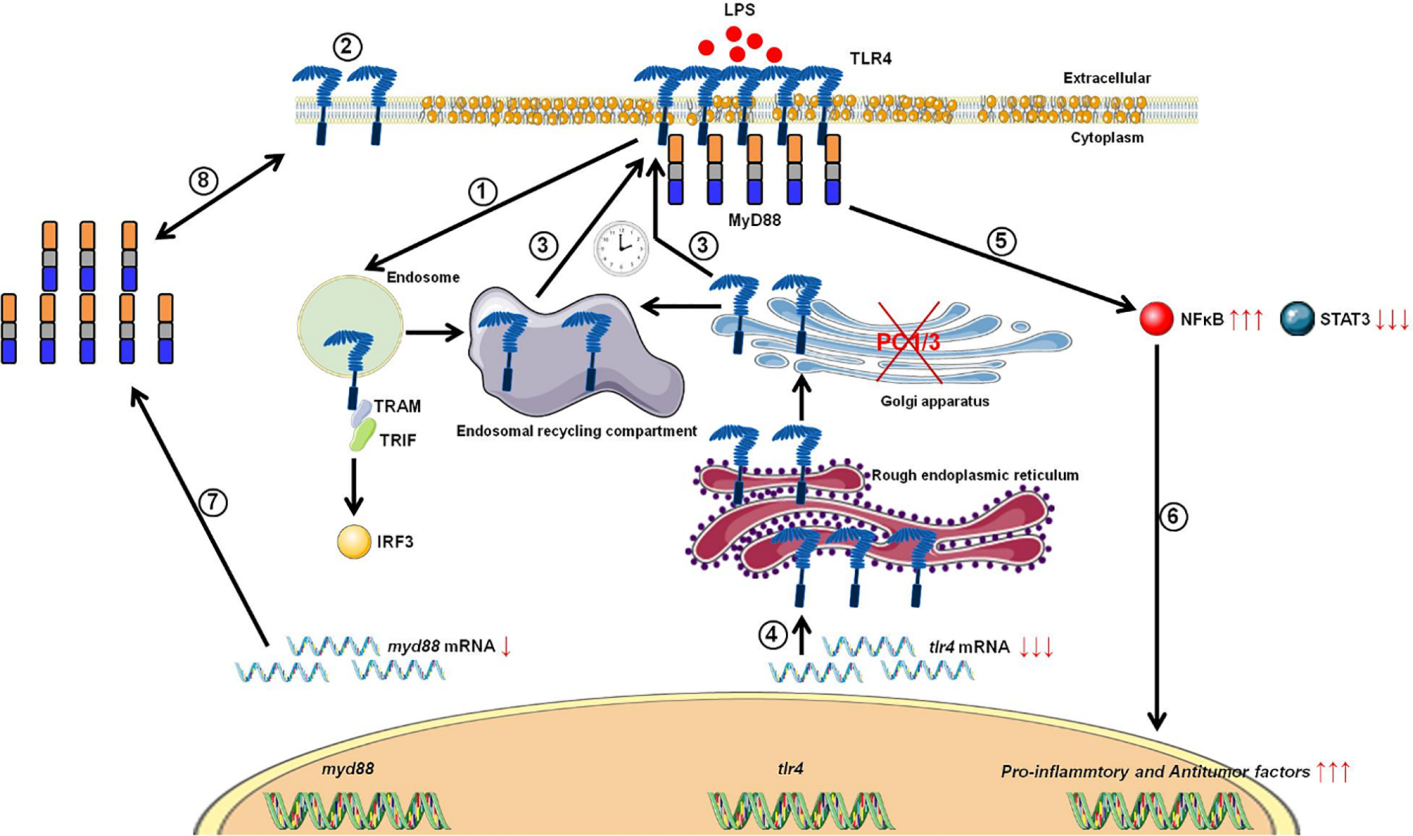
Proposed model depicting the impact of PC1/3 knockdown on the activation of TLR4. Yellow droplets in the cell membrane symbolize that KD cells exhibited modifications of their lipid profile. (1) After LPS treatment, TLR4 is internalized in the endosomes to trigger the MYD88-independentsignaling resulting in the activation of IRF-3. (2) As a result, the level of TLR4 at the plasma membrane decreases. (3) In the absence of PC1/3, TLR4 is more quickly re-expressed at the cell surface as symbolized by the clock pictogram, *i.e*,. 3 h after LPS treatment. These receptors may translocate from endosomal recycling compartment (ERC) where they are stored or from the Golgi apparatus after their synthesis and maturation. (4) TLR4 receptor can be synthesized from a pre-existing pool of messengers. This may support the *de novo* expression of the receptor at the cell surface or the replenishment ofTLR4 stock in ERC if the receptor translocated from this compartment. (5) The re-expression of TLR4 at the cell surface after 3 h of LPS challenge in PC1/3 KD macrophages leading to a stronger activation of the pro-inflammatory NFKB pathway while the anti-inflammatory STAT3 pathway is downregulated. (6) As a consequence, PC1/3 KD macrophages stimulated with LPS secrete more pro-inflammatory cytokines and antitumor factors. After treatment of PC1/3 KD cells with LPS during 6 h, TLR4 is again internalized, and its amount at the cell surface decreased (2). (7) The increase of MYD88 level observed in PC1/3 KD macrophages after 6 h of LPS treatment may compensate the decrease of TLR4 at the cell surface and maintain the activation of the signaling pathway. (8) A clear correlation between protein synthesis and gene expression in KD cells treated with LPS cannot be made. Therefore, another mechanism, such as augmentation of protein half-life or stability may be responsible for this increase. (9) PC1/3 KD cells resist to the inhibitory effect of IL-10 and clearly show a pro-inflammatory response.

**Figure 4:**
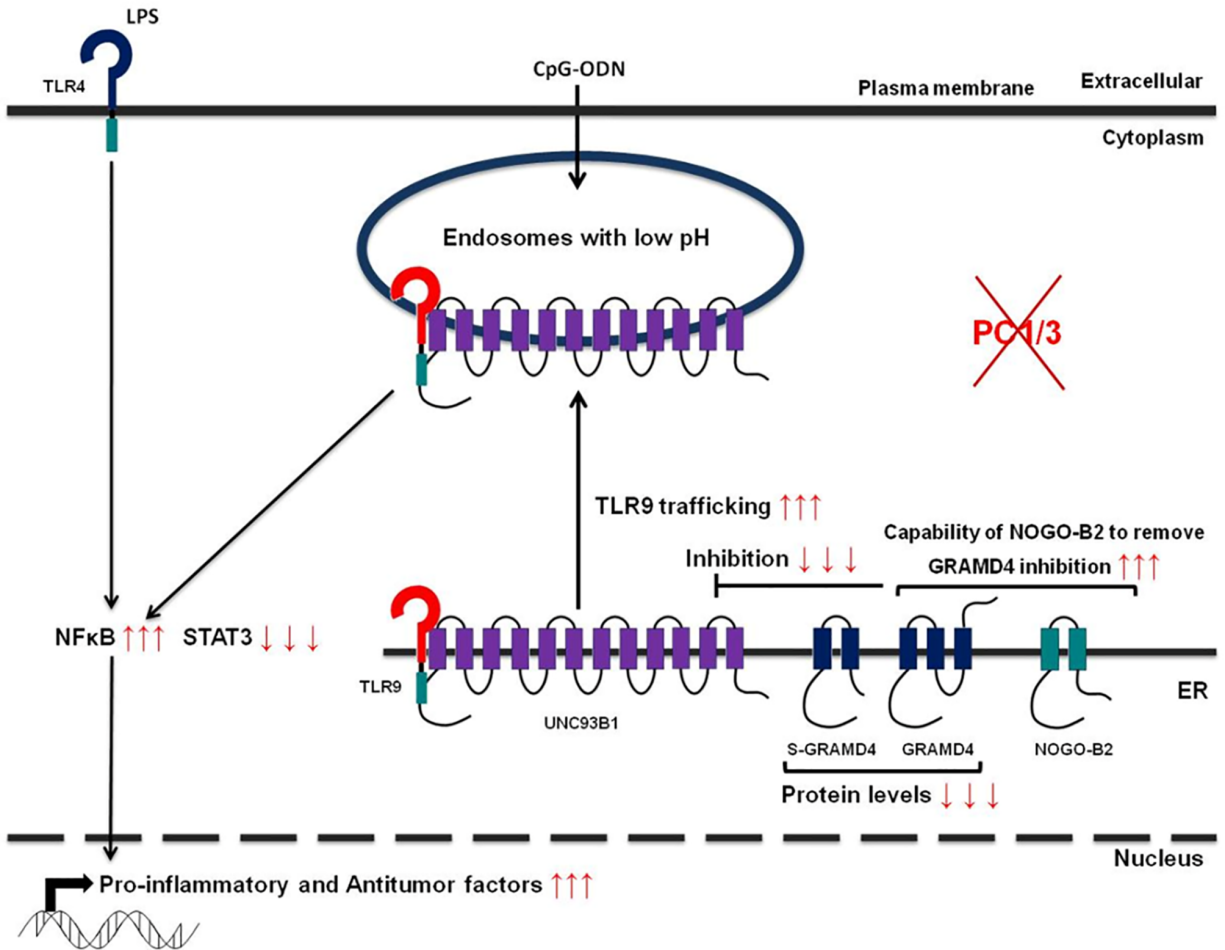
Schematic depicting how PC1/3 knockdown impacts TLR4 and 9 activation in macrophages.

Studies from our group have also demonstrated that PC1/3 regulates TLR activity in macrophages. Following LPS stimulation, PC1/3 trafficked with TLR4 (27). Of note, PC1/3 knock down led to a quicker recycling of TLR4 at the cell surface (36) (**Figure 3**). This revealed an essential role of PC1/3 in TLR4 trafficking. An over-expression of MYD88 was also observed in PC1/3 KD macrophages treated with LPS (36) (**Figure 3**). As a consequence, a longer activation of the NF-κB signaling pathway was observed (28). Similarly, TLR9 trafficking was also shown to be directly linked to PC1/3 (29) through the regulation of GRAMD4 levels (37) (**Figure 4**). In PC1/3 KD macrophages treated with CpG-ODN, TLR9 clustered in multivesicular bodies leading to a quicker activation of the NF-κB signaling pathway (29). All these results showed that PCs, especially furin and PC1/3, are central in the processing, maturation and biological activity of TLRs across cell types and species. However, more studies are needed to decipher their exact role in TLR trafficking.

## MHC-I and PCs

The major histocompatibility complex I (MHC-I) is essential for antigen presentation and immune response (38). MHC-I ligands are mainly produced by proteasomes. However, part of MHC-I ligands are also generated in the trans-Golgi network. Interestingly, it was demonstrated that in this compartment furin was responsible for the proteolytic cleavage and liberation of antigenic peptides (39, 40). Another study revealed that PC7 could be also a key regulator of antigen presentation since it was required for stable MHC-I expression at the cell surface (41).

Since PCs regulate various functions in immune cells they may constitute potent therapeutic targets for the effective treatment of immune diseases or pathologies involving immune cells such as cancers.

## PCs in Oncoimmunotherapy

The role of PCs in oncology has been widely studied and their relationship with tumor progression has been demonstrated. PCs are known to be over-expressed in several types of cancers such as PC1/3 and PC2 in pituitary adenomas (42), PACE4 in ovarian (43) and prostate cancers (44). An increase in furin expression, related to the invasiveness of the tumor, was also observed in several head and neck cancers (45), especially in gliomas (46). Besides, PCs also appeared to contribute to tumor development through cleavage and activation of substrates involved in various biological processes. For example, cell proliferation is most likely increased by the activation of growth factors such as platelet-derived growth factor (PDGF), insulin-like growth factor (IGF) and transforming growth factor (TGF-β). All of these factors are processed by PCs (3, 47). PCs also contribute to the maturation of the vascular endothelial growth factor (VEGF) and some enzymes involved in the degradation of the extracellular matrix such as matrix metalloproteinases (MMPs). Thus they promote angiogenesis and metastasis development (3, 47). Some PCs, such as furin, are also known to cleave integrins which control cell mobility and thereby support the metastatic process (48).

Considering the involvement of PCs in tumor development, inhibition of their proteolytic activity appears to be a new therapeutic approach. Thus, several inhibitors of PCs have been developed to limit tumor development (49). Their anti-tumor effect has been demonstrated in colorectal cancer (50) and prostate cancer (51). The human colorectal cancer cell line HT-29 treated with α1-antitrypsin Portland (α1-PDX), a PCs inhibitor, showed a decrease in TNF-α and IL-1α secretion and a lack of IGF-1R maturation. Also, the administration of a PCs inhibitor in a mouse model of carcinoma led to a reduction in tumor size (50). Moreover, Prof. R. Day and colleagues showed that the injection of a PCs inhibitor significantly decreased tumor progression in a mouse model of prostate cancer (51).

In addition to their direct inhibitory roles in cancer cells, PCs can also be good candidates to reactivate immune cells within the tumor microenvironment. Immune cells are part of the tumor microenvironment (TME) and the dialogue between immune cells and the TME plays a central role in tumor development. Among them, macrophages are the most abundant immune cells of the TME. Macrophages are educated by the tumor cells to participate to its progression and propagation. Tumor-associated macrophages are involved in numerous pro-tumoral functions such as angiogenesis, invasion and metastasis formation. They also regulate the anti-tumoral functions of other immune cells and decrease the efficacy of chemo- and radiotherapies. The central role of macrophages in tumor development makes them ideal therapeutic targets. Most immunotherapeutic approaches in cancer focus on cytotoxic T cell reactivation through, for example, the inhibition of the immune checkpoints PDL1 and CTLA-4. Their efficacy is based on the capacity of T cells to infiltrate the TME and on the development of a favorable environment for cytotoxic responses. However, these conditions are often not fulfilled due to a high macrophages abundance which creates an immunosuppressive environment. Some studies have demonstrated that the resistance to the immune checkpoint therapies can be circumvented by depleting macrophages from the TME with a CSF1R inhibitor (52–54). We have also to keep in mind that macrophages can promote an anti-tumoral cytotoxic response. Therefore, therapies could benefit more from strategies that reprogram macrophages from a pro- to an anti-tumoral state, rather than those aiming at eliminating them. For example, cytokines, Toll-like receptor agonists and CD40 agonists have all been reported to induce repolarization of tumor-associated macrophages (55–57). Genetic reprogramming of macrophages is another option under investigation (58).

**Figure 5:**
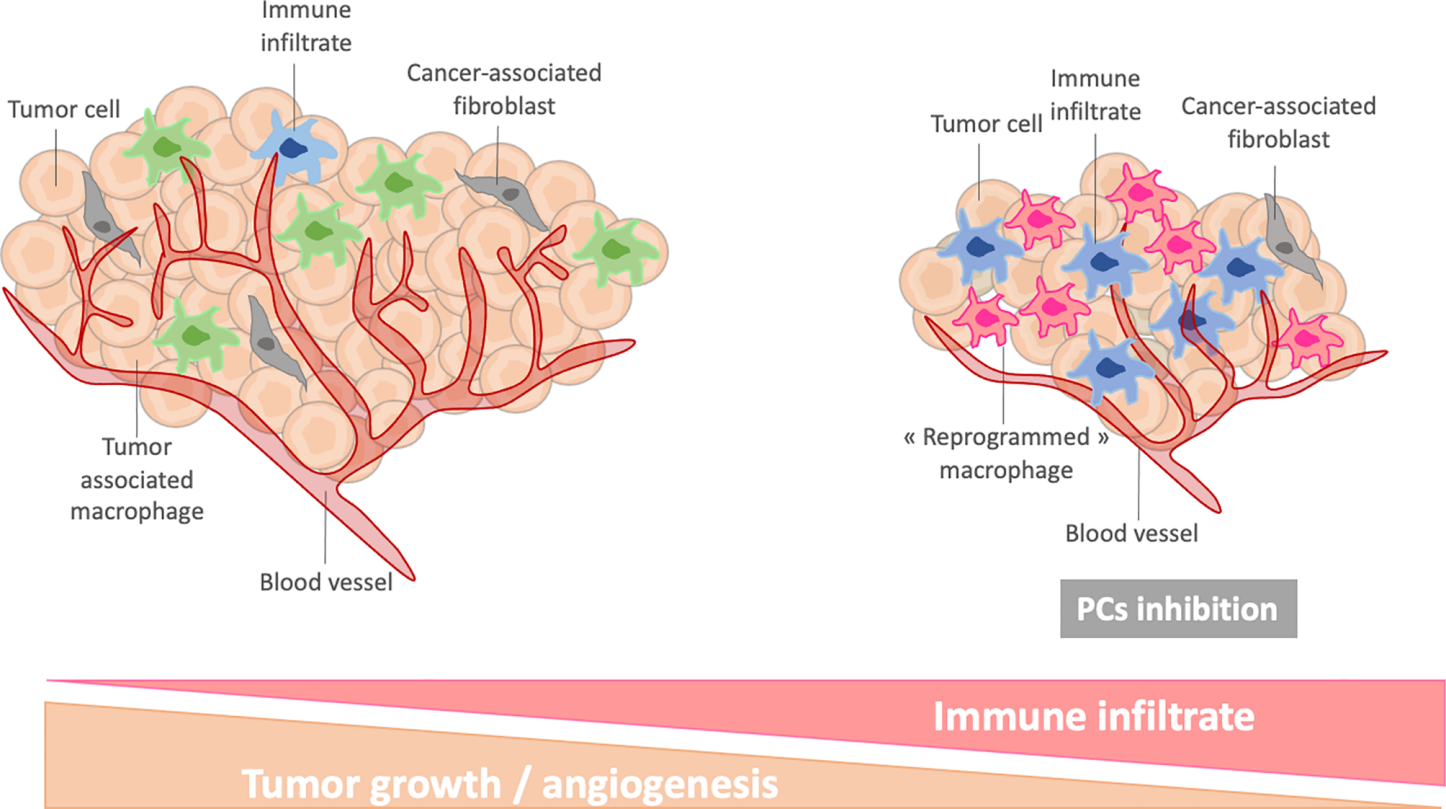
Schematic representation of reprogrammed macrophages after PC inhibition in tumor microenvironment.

On the other hand, the involvement of PCs in macrophages phenotype regulation makes them good candidates to reprogram macrophages in an anti-cancer therapeutic perspective. In our previous studies, we have shown that the massive secretion of immune factors by PC1/3 KD macrophages stimulated with LPS could have interesting biological effects. In fact, these secreted factors had a strong chemotactic activity on T lymphocytes which can be attributed to the chemokines CXCL10, CXCL9 and CCL20 (28). Moreover, LPS stimulation of PC1/3 KD macrophages triggered the secretion of TNF-α, IL-1α and IL1-β which are important factors involved in T lymphocytes activation. PC1/3 KD macrophages could therefore increase the recruitment of activated T lymphocytes in the TME leading to increase cytotoxicity. In addition to attracting lymphocytes, the PC1/3 KD secretome had also a direct anti-tumoral activity on breast and ovarian cancer cells. Moreover, several proteins involved in the phagocytic activity of macrophages were over-expressed in PC1/3 KD macrophages (28). An interesting fact is that PC1/3 KD macrophages were resistant to the anti-inflammatory cytokine IL-10 which is predominant in the TME (28, 31). Similar results were obtained with Paclitaxel, a chemotherapeutic drug and TLR4 agonist (31). PC1/3 KD macrophages stimulated with Paclitaxel secreted high levels of cytokines and chemokines which had anti-tumoral activities on glioma cells. Therefore, PC1/3 inhibition coupled to the use of TLR agonists could be an interesting therapeutic opportunity to counteract efficiently the tumoral immunosuppression. In this way, several studies have shown the potential use of a PCs inhibitor as an anti-tumoral immunotherapy. Indeed, macrophages treated with a PCs inhibitor were programmed toward a pro-inflammatory phenotype. They also expressed and secreted less pro-tumoral factors. This was observed even in the presence of the immunosuppressive TME in co-cultured spheroids (32). The PCs inhibitor used in this study had direct anti-tumoral activities on glioma cells which were retained in presence of tumor-associated macrophages. The effects of the PCs inhibitor were even more intense when combined with a TLR3 agonist (59). In another study, inhibition of PCs repressed PD-1 expression and enhanced CD8 lymphocytes infiltration in colorectal tumors (60). Thus, targeting PCs in immune cells could provide a novel strategy to both reprogram tumor-associated macrophages and promote cytotoxic T cell response. The reprogrammation of tumor-associated macrophages by PCs inhibition has been shown to stably decrease their immunosuppressive properties. Therefore, they could be important to enhance T lymphocytes activities (**Figure 5**). Such a strategy can also be used to increase the efficacy of current chemotherapies since tumor-associated macrophages can interfere with their efficacy. It can also boost the development of immunotherapies for solid tumors such as chimeric antigen receptor strategies or immune checkpoints inhibition which are very limited due to the immunosuppressive environment.

Targeting proprotein convertases in anti-cancer therapeutic strategies is novel. Thus, currently, very few clinical trials exist. A phase I clinical trial has recently assessed the toxicity and response to intradermal injection of a vaccine in patients with advanced cancers (61). This vaccine included a plasmid encoding granulocyte-macrophage colony-stimulating factor (GMCSF) and a bifunctional short hairpin RNAi (bi-shRNAi) targeting furin. Following these results, a Phase II clinical trial was performed. It demonstrated that ovarian cancer patients who received this vaccine displayed an increased T cell activation and improved recurrence-free survival (RFS) (62). These results justify the initiation of a phase III clinical trial (ClinicalTrials.gov Identifier: NCT02346747) (63).

Proprotein convertases are also targeted for the treatment of other diseases, especially PCSK9 in cardiovascular diseases. Thus a phase III clinical trial demonstrated that inhibition of PCSK9 in pediatric patients with heterozygous familial hypercholesterolemia reduced the LDL cholesterol level and other lipid variables (ClinicalTrials.gov Identifier: NCT02392559) (64).

## Conclusion

Proprotein convertases are proteases involved in many biological processes and are essential in the regulation of the immune system. Especially, furin, PC1/3, PC5/6 and PC7 regulate the mechanisms of antigen presentation and many other functions of macrophages and lymphocytes. However, it is necessary to complete the various studies to decipher the exact role of these enzymes within the immune system.

## Ethics Statement

The studies involving human participants were reviewed and approved by National Medical Ethics Committee, Republic of Slovenia, Ministry of Health (reference number 0120-88/2020/3). The patients/participants provided their written informed consent to participate in this study.

## Author Contributions

Conceptualization, MS. Writing-original draft preparation, MR, MD, MS. Writing-review and editing, MR, MD, FR, MS. Supervision, MS. All authors contributed to the article and approved the submitted version.

## Funding

This research was supported by the funding from Ministère de l’enseignement Supérieur, de la Recherche et de l’innovation(MESRI), Institut National de la Santé et de la Recherche Medical Research (Inserm), Hauts-de-France region and Université de Lille.

## Conflict of Interest

The authors declare that the research was conducted in the absence of any commercial or financial relationships that could be construed as a potential conflict of interest.
